# *Ac*-HSP20 regulates autophagy and promotes the encystation of *Acanthamoeba castellanii* by inhibiting the PI3K/AKT/mTOR signaling pathway

**DOI:** 10.1186/s13071-024-06436-w

**Published:** 2024-08-19

**Authors:** Siyao Guo, Di Liu, Xi Wan, Dingrui Guo, Meiyu Zheng, Wenyu Zheng, Xianmin Feng

**Affiliations:** 1 Department of Pathogenic Biology, Jilin Medical University, Jilin, China; 2 Department of Microsurgery, Jilin City Central Hospital, Jilin, China; 3 Department of Clinical Laboratory, Jilin City Hospital of Chemical Industry, Jilin, China

**Keywords:** *Acanthamoeba castellanii*, Encystation, Autophagy, HSP20, PI3K/AKT/mTOR, Protozoa

## Abstract

**Background:**

The encystation of *Acanthamoeba castellanii* has important ecological and medical significance. Blocking encystation is the key to preventing transmission and curing infections caused by *A. castellanii*. The formation of autophagosomes is one of the most important changes that occur during the encystation of *Acanthamoeba*. Our previous studies have shown that the heat shock protein 20 of *A. castellanii* (*Ac*-HSP20) is involved in its encystation. This study aimed to determine the role and mechanism of *Ac*-HSP20 in regulating autophagy involved in the encystation of *A. castellanii.*

**Methods:**

Immunofluorescence assay, western blotting and transmission electron microscopy were used to analyze the dynamic changes in autophagy during the initiation and continuation of encystation. The knockdown of *Ac*-HSP20 was performed to clarify its regulation of encystation and autophagy and to elucidate the molecular mechanism by which *Ac*-HSP20 participates in autophagy to promote cyst maturation.

**Results:**

The encystation rates and autophagosomes were significantly decreased by treatment with the autophagy inhibitor 3-MA. The autophagy marker LC3B and autophagic lysosomes increased with the induced duration of encystation and reached the maximum at 48 h. The encystation rate, LC3B expression and autophagosomes decreased when *Ac*-HSP20 was knocked down by siRNA transfection. In addition, the expression levels of *Ac*-HSP20 and LC3B increased and the expressions of p-AKT and p-mTOR decreased after 48 h of encystation without knockdown. However, the expressions of p-AKT and p-mTOR increased while the expression of LC3B decreased under the knockdown of *Ac*-HSP20. Furthermore, the protein expression of LC3B increased when the PI3K/AKT/mTOR signaling pathway was inhibited but decreased when the pathway was activated.

**Conclusions:**

The results demonstrated that autophagy is positively correlated with the encystation of *A. castellanii*, and *Ac*-HSP20 regulates autophagy to maintain the homeostasis of *A. castellanii* by inhibiting the PI3K /AKT /mTOR signaling pathway, thus promoting the maturation and stability of encystation.

**Graphical Abstract:**

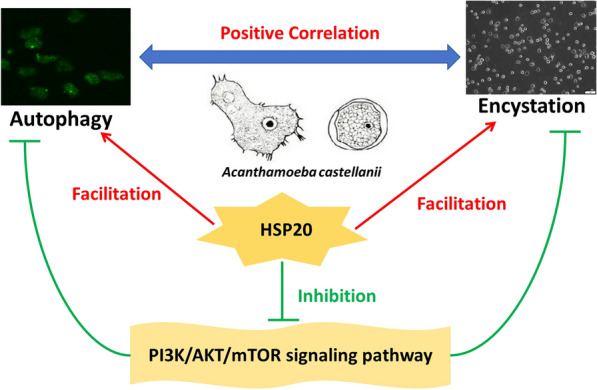

## Background

Encystation is a common response to environmental stress in most protists, including free-living amoebas. The term encystation refers to the transformation of active foraging trophozoites into dormant cysts or similar forms, such as spores, dormant spores, oospores or zygotes, in inhospitable environments [1, 2]. In addition to ensuring survival, cyst formation represents an important way for organisms to spread into the environment. The encystation of protozoan parasites such as *Entamoeba histolytica*, *Giardia lamblia*, *Toxoplasma gondii* and *Acanthamoeba castellanii* (*A. castellanii*), etc., is closely related to human health [3–5]. Cysts are formed when these parasites are stimulated by changes in the parasitic environment, such as drug concentrations, immune risk and abnormal parasitism. However, drug resistance, chronic infection and recurrence are made possible by the extreme resistance of the cyst to various antimicrobial agents, disinfectants, dryness and radiation [6]. Therefore, the encystation of protozoa is an important scientific issue of international concern due to both its ecological importance and the serious risks that cysts pose to human health.

The genus *Acanthamoeba* contains representative protozoa that are widespread in nature as free-living organisms. *Acanthamoeba* is characterized by opportunistic pathogenicity, and protozoa in this genus can invade through damaged skin, the ocular conjunctiva and urogenital tract to infect immunocompromised individuals, causing blindness (*Acanthamoeba* keratitis) and granulomatous amebic encephalitis [7, 8]. The free-living *Acanthamoeba* can also parasitize human body through sexual transmission [9]. The life cycle of *A. castellanii* consists of two stages: the trophozoite stage, which is characterized by replication and nutrient uptake, and the cyst stage, which is characterized by a double-walled structure and a dormant state. The trophozoites transform into cysts when encountering adverse factors such as immune resistance, nutritional deprivation and chemotherapy [7]. For free-living amoebas, the cysts are also formed in response to changes in the external environment such as temperature variations and pH stress [10]. Cysts can effectively resist the invasion of environmental factors and may survive in the natural environment and at the local infection site for several years, which is the fundamental reason underlying the widespread occurrence, chronicity, difficult radical treatment and recurrence of *Acanthamoeba* infections [11]. In addition, *Acanthamoeba* feeds on bacteria through phagocytosis, providing shelter for bacterial pathogens such as *Legionella pneumoniae*, *Vibrio cholerae*, *Mycobacterium leprae* and methicillin-resistant *Staphylococcus aureus*, which are retained in the cysts, causing *Acanthamoeba* to become an important vector for the survival and transmission of these pathogens [12]. Blocking encystation is the key to controlling the transmission of *Acanthamoeba* and the pathogens it carries and to curing *Acanthamoeba* infections. Therefore, clarifying the mechanism of encystation is the key and will provide a foundation for further research on controlling *Acanthamoeba*.

The encystation of protozoa is regulated by many factors, such as the food source, culture environment, external stress and age [13]. In the absence of an external material supply, the protozoan completes a series of complex processes through self-regulation. Autophagy is a cellular homeostatic process that degrades damaged organelles and macromolecules through lysosomes, which is a unique phenomenon in eukaryotic cells [14]. Cells degrade their cytoplasmic proteins and organelles using lysosomes in the form of vacuoles, thus eliminating cellular senescence-damaged organelles and accumulated denatured proteins, initiating the recycling of amino acids under starvation conditions [15]. The formation of autophagosomes is one of the most important changes in the encystation of *Acanthamoeba* [16, 17]. The Sir2 gene of *Acanthamoeba* also plays a crucial role in this process [18]. However, the autophagy mechanism related to the encystation of *Acanthamoeba* remains unclear.

The heat shock protein (HSP) family is a class of important molecular chaperones, most of which are ATP-dependent proteins that are expressed during cell proliferation [19]. A previous study on the drug sensitivity of *Schistosoma mansoni* showed that HSP70 enabled the parasite to survive the killing effect of drugs by inhibiting apoptosis and inducing autophagy [20]. The HSP90 inhibitor 17-AAG can abnormally activate the autophagy pathway of *Leishmania*, resulting in ultrastructural alterations of the parasite, formation of immature autophagosomes and, ultimately, the death of *Leishmania* [21]. The HSP family can affect the physiological metabolism of protozoa through autophagy. In our previous work, the relationship between HSP20 of *A. castellanii* (*Ac*-HSP20) and encystation was analyzed. We found that *Ac*-HSP20 is an important molecule that is involved in the regulation of encystation and is related to the initiation of the transformation of trophozoites into cysts [22]. In addition, the autophagosomes and autophagolysosomes increased significantly after the induction and maturation of cysts. It is inferred that the encystation of *A. castellanii* consists of two processes: the initiation of encystation regulated by HSPs and maturation of cysts promoted by cell homeostasis related to autophagy. Therefore, the interaction of HSPs and autophagy is the key to revealing the mechanism of encystation in *A. castellanii*. Relevant research was performed to verify the above speculation in this study.

## Methods

### Reagents and chemicals

*Acanthamoeba castellanii* (ATCC30011) and *Escherichia coli* (ATCC29552TM) were obtained from ATCC. Gentamycin sulfate was purchased from Yuanye Biotech (Shanghai, China). Protein Marker was obtained from Thermo Fisher Scientific (Waltham, MA, USA). Agar powder, sodium dodecyl sulfate (SDS), Tris-base and glycine were obtained from Dingguo Biotech (Beijing, China). 3-Methyladenine (3-MA) was obtained from Bio-Rad (Hercules, CA, USA). Fetal bovine serum (FBS) was obtained from Biological Industries (BI, Israel). Rabbit and mouse antibodies against LC3B (18725-1-AP), AKT (60203-2-lg), mTOR (66888-1-lg), p-AKT (66444-1-lg), p-mTOR (67778–1-lg) and β-actin (66009-1-lg) were from Proteintech (Wuhan, China). Polyvinylidene difluoride (PVDF) membranes were obtained from Millipore Corporation (Billerica, MA, USA). LB medium, 4% paraformaldehyde, anti-fluorescence quenching sealing liquid, Triton X-100, RNAeasy Animal RNA Isolation Kit (Spin Column), II First Strand cDNA Synthesis Kit with gDNA Eraser, 8% SDS-PAGE gel, Tris-glycine buffer, total protein (based BCA method), DIPA dye, goat anti-rabbit antibodies and enhanced ECL reagent were obtained from Beyotime Biotech (Shanghai, China). SYBR SuperMix Plus was obtained from Novoprotein Scientific Inc. (Shanghai, China). Yeast extract and proteose peptone were purchased from Sigma (St. Louis, MO, USA). Lipofectamine 3000 reagent was purchased from Invitrogen (Carlsbad, CA, USA). *Ac*-HSP20 siRNA reagents and negative control siRNA were obtained from GenePharma (Shanghai, China). PI3K/mTOR Inhibitor-11 (HY-151622) and Recilisib (HY-101625) were purchased from MCE (NJ, USA).

### *Acanthamoeba castellanii* culture

*Acanthamoeba castellanii* were cultured in peptone-glucose-yeast extract (PGY) medium (pH 6.5) containing 2% (w/v) proteose peptone, 0.1% (w/v) yeast extract, 0.8% (v/v) 0.05 mol/l CaCl_2_, 1%(v/v) 0.4 mol/l MgSO_4_ · 7H_2_O, 1% (v/v) 0.25 mol/l Na_2_HPO_4_ · 7H_2_O, 0.1% (w/v) NaCitrate · 2H_2_O and 1% (v/v) 0.005 mol/l Fe(NH_4_)_2_(SO_4_)_2_ · 6H_2_O, supplemented by 5% (w/v) 2 mol/l glucose and 50 μg/ml gentamicin, in a sterile incubator at 25 °C for 3–5 days [10, 22]. The cultured trophozoites were co-cultured with inactivated *E. coli* for 5–7 days, which served as food for the trophozoites [10, 23]. The trophozoites were collected when they entered the logarithmic growth phase.

### Dynamic observation of encystation in *A. castellanii*

The cyst-forming medium was improved according to a previous study with some modifications [10]. The trophozoites were cultured in a cyst-forming medium containing 0.555% (w/v) 95 mmol/l NaCl, 0.0372% (w/v) 5 mmol/l KCl, 0.197% (w/v) 8 mmol/l MgSO_4_, 0.0044% (w/v) 0.4 mmol/l CaCl_2_, 0.004% (w/v) 1 mmol/l NaHCO_3_ and 0.121% (w/v) 20 mM Tris–HCL. *Acanthamoeba castellanii* culture grown in PGY medium was inoculated into the cyst-forming medium to give approximately 3 × 10^6^ cells ml^−1^ and placed on ice to induce cyst formation for 0, 24, 48 and 72 h at 0 °C, respectively [22]. Cells stopped dividing but remained viable and thereafter began to encyst. The morphological changes and vitality of *A. castellanii* were observed with an inverted microscope. The numbers of trophozoites and cysts were determined by hemocytometer slide [24]. The encystation rate of *A. castellanii* trophozoites was calculated by the ratio of cysts to total *A. castellanii*. Samples of total RNA and total protein were collected at each time point for further study.

### Identification of autophagy marker LC3B by immunofluorescence (IF) staining

Immunofluorescence staining was performed as described in our previous study [22] with some modification to detect the expression of LC3B. Briefly, the *A. castellanii* were cultivated on cover slips treated with 4 μg/cm^2^ Cell-Tak in six-well plates until the density of the trophozoite was 80%. The cover slips were washed with pre-cooled PBS solution twice for 3 min each time, followed by fixation with 4% paraformaldehyde solution for 30 min. After washing with pre-cooled PBS solution twice, the cover slips were blocked with 5% calf serum at room temperature for 1 h and then incubated with LC3B antibody diluted with 1% bovine serum albumin (1:500) at 4 °C overnight in the dark. After washing with PBS, the cover slips were incubated with Alexa Fluor 488-conjugated anti-rabbit IgG secondary antibody (1:500) at 37 °C in the dark for 1 h. After incubation, the *A. castellanii* were stained with DAPI solution for 20 min and 150 nM Lyso Tracker Red for 1 h at room temperature followed by a tablet seal with anti-fluorescence quenching. Finally, the *A. castellanii* were observed using a laser scanning confocal microscope (Olympus, Japan). ImagePro Plus 6.0 software (US National Institutes of Health, Bethesda, MD, USA) was used to quantify the area of enrichment region in each image and the fluorescence IOD value. The relative intensity of LC3B in three independent experiments was measured by the ratio of total integrated optical density (IOD) to area.

### Quantification of PI3K/AKT/mTOR signaling pathway by western blotting

After centrifugation at 2000 r/min at 4 °C for 10 min, the *A. castellanii* were collected and lysed with RIPA lysate containing 1% protease inhibitor for 20 min on ice and then sonicated for 5 min. The protein in the supernatant was collected after centrifuging at 12,000 r/min at 4 °C for 15 min. The concentration of protein was determined using BCA kit according to the manufacturer’s instructions. After denaturation at 100 °C for 10 min, protein fractions (30 μg) were isolated by 12% SDS-PAGE gel electrophoresis and transferred onto PVDF membranes. The PVDF membranes were blocked with 5% (w/v) skim milk for 1 h at room temperature and then incubated with LC3B (1:1000), AKT(1:1000), mTOR (1:1000), p-AKT (1:1000), p-mTOR (1:1000) and β-actin (1:2000) antibodies overnight at 4 °C. After washing with TBST, the membranes were incubated with HRP goat anti-rabbit IgG (1:3000) and HRP goat anti-mouse IgG (1:3000) for 1 h at 37 °C. The protein bands were developed with ECL luminescent agent and analyzed using Image J software. The experiment was repeated three times.

### Observation of the autophagosomes during encystation by transmission electron microscopy (TEM)

After centrifugation at 2000 r/min for 10 min, the *A. castellanii*, treated with an ice bath for 0, 24, 48 and 72 h, were collected and re-suspended with 1 ml sterilized PBS solution. The *A. castellanii* were transferred into 2-ml centrifuge tubes and centrifuged at 1000 rpm for 5 min, and this was repeated twice. After centrifugation, the *A. castellanii* were fixed with glutaraldehyde at 4 °C for 30 min, followed by dehydration, embedding and ultra-thin sectioning. Finally, the morphology of autophagosomes in each group was observed and photographed under TEM (JEOL, Japan). All the experiments were repeated independently three times.

### Knockdown of HSP20 by siRNA

The trophozoites of *A. castellanii* were cultivated in a 6-cm dish until the density was 80%. The trophozoites were transfected with FAM fluorescently labeled HSP20-specific siRNA (forward sequence 5′-CCGAUCCCUGGUCUGACAUTT-3′ and reverse sequence 3′-AUGUCAGACCAGGGAUCGGTT-5′) using Lipofectamine 3000 reagent following the manufacturer’s directions. In the negative control group, there was no interfering sequence siRNA in the trophozoites. The transfection efficiency was observed by fluorescence microscopy after 6 h; RT-PCR and western blot were performed 24 h and 48 h after transfection, respectively.

### Identification of the transcriptional inhibition of HSP20 by qRT-PCR

Briefly, total RNA samples from the cultured *A. castellanii* were extracted using RNA Isolation Kit following the manufacturer’s instructions. Total RNA (75 µg) was synthesized into cDNA using a reverse transcription cDNA synthesis kit. The primers of HSP20 (GenBank: MT323119.1) and β-actin (GenBank: XM_004352791.1) were selected using Primer Premier 5.0 software. The sequences of the primers were: β-actin (forward primer 5′- GTATGCTCCTCCTCAAG -3′, reverse primer 5′- TAGAAGGTGTCCATCCA -3′) and HSP20 (forward primer 5′- ACATGGACGTGCGTGAG -3′, reverse primer 5′- AAGCGTGCGCTTGAAGG -3′). The PCR reaction system was prepared according to the instructions of the SYBR PCR kit and placed in the real-time PCR system (Thermo, Waltham, MA, USA). The conditions were set as follows: 95 °C for 10 min, followed by 30 cycles of 95 °C for 30 s, 60 °C for 60 s and 72 °C for 60 s. The relative gene expression was calculated using the comparative Ct method formula: 2^−ΔΔCt^.

### Statistical analysis

Fluorescence quantitation and grayscale analysis were performed using Image J. Histogram graphs and statistical analyses were performed using GraphPad Prism 8.0.1. Measurement data were expressed as means ± SD. Statistical analysis between two groups was performed using the t-test. Between multiple groups, one-way ANOVA was performed. The difference was statistically significant at *P* < 0.05.

## Results

### Autophagy of *A. castellanii* was related to encystation

**Fig. 1:**
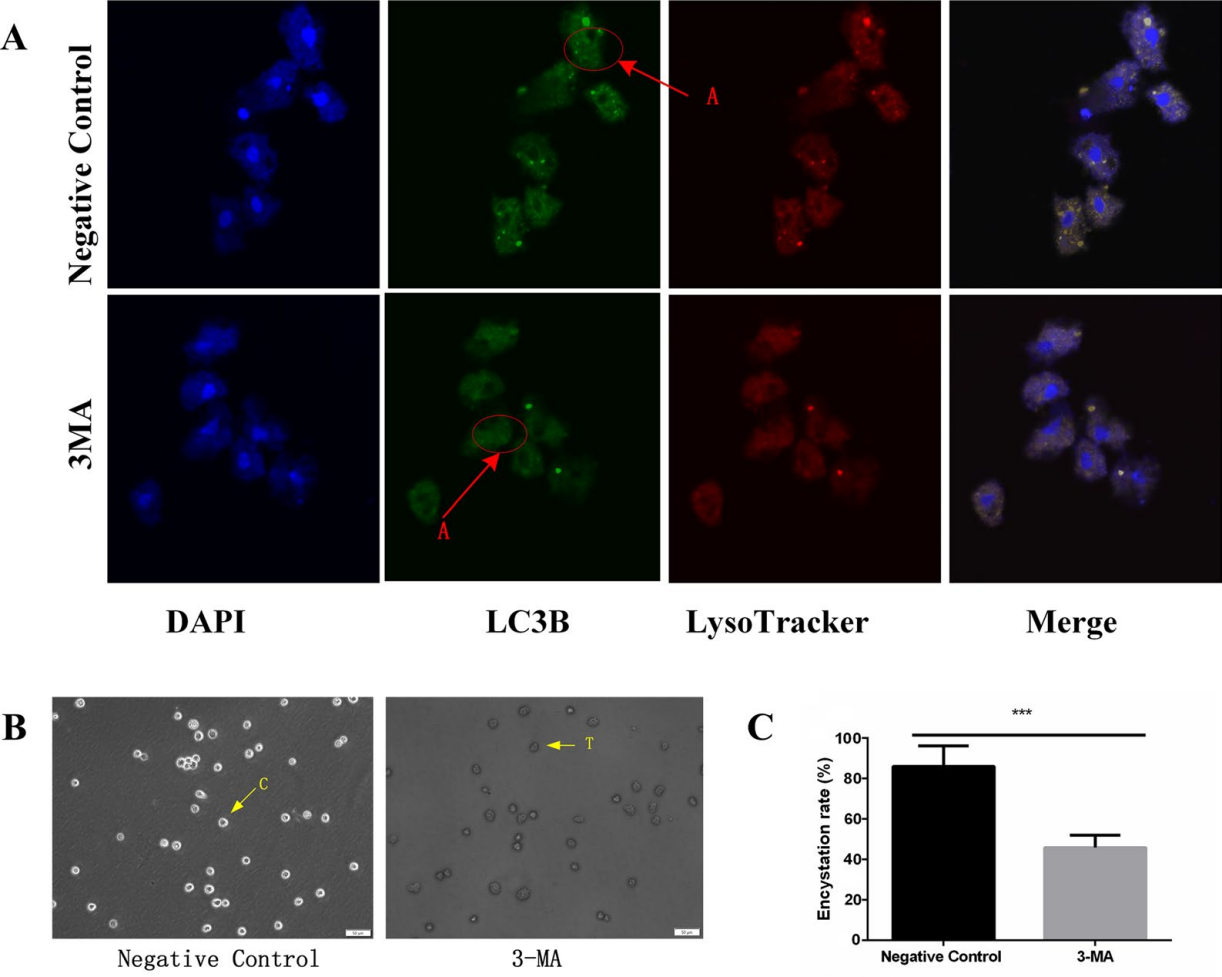
Effects of 3-MA treatment on autophagy and encystation rate of *Acanthamoeba castellanii.* The trophozoites were pretreated with 3-MA, and encystation was induced under ice bath for 48 h. **A** Fluorescence signal of autophagy protein LC3B after treatment with 3-MA (5 mmol/l) (A, the typical fluorescent spots of autophagic vesicles formed after the occurrence of autophagy in *A. castellanii*). DAPI-treated nucleus of the *A.castellanii* showed blue fluorescence. LysoTracker Red-treated lysosomes of the *A. castellanii* showed red fluorescence. The cytoplasm of *A. castellanii* showed green fluorescence after treatment with primary anti-LC3B antibody and secondary anti-Alexa Fluor® 488 green fluorescent dye. **B** Encystation after 3-MA treatment was observed by optical microscope (T = trophozoite, C = cyst). **C** Change of encystation rate after 3-MA treatment (3 fields were randomly selected, and the average encystation rate from three fields was calculated. ***P < 0.001)

The results of the IF assay showed that the fluorescence intensity of LC3B, an autophagy marker, in the 3-MA inhibitory group was significantly reduced compared with the control group (Fig. 1A), indicating that 3-MA treatment successfully inhibited autophagy in *A. castellanii*. In addition, the encystation rate of *A. castellanii* in the 3-MA inhibitory group was 43.43%, which was significantly lower than that in the control group (83.61%, *P* < 0.001, Fig. 1B, C), indicating that the encystation rate in *A. castellanii* was significantly decreased when autophagy was inhibited.

### Analysis of the relationship between autophagy and encystation in *A. castellanii*

**Fig. 2:**
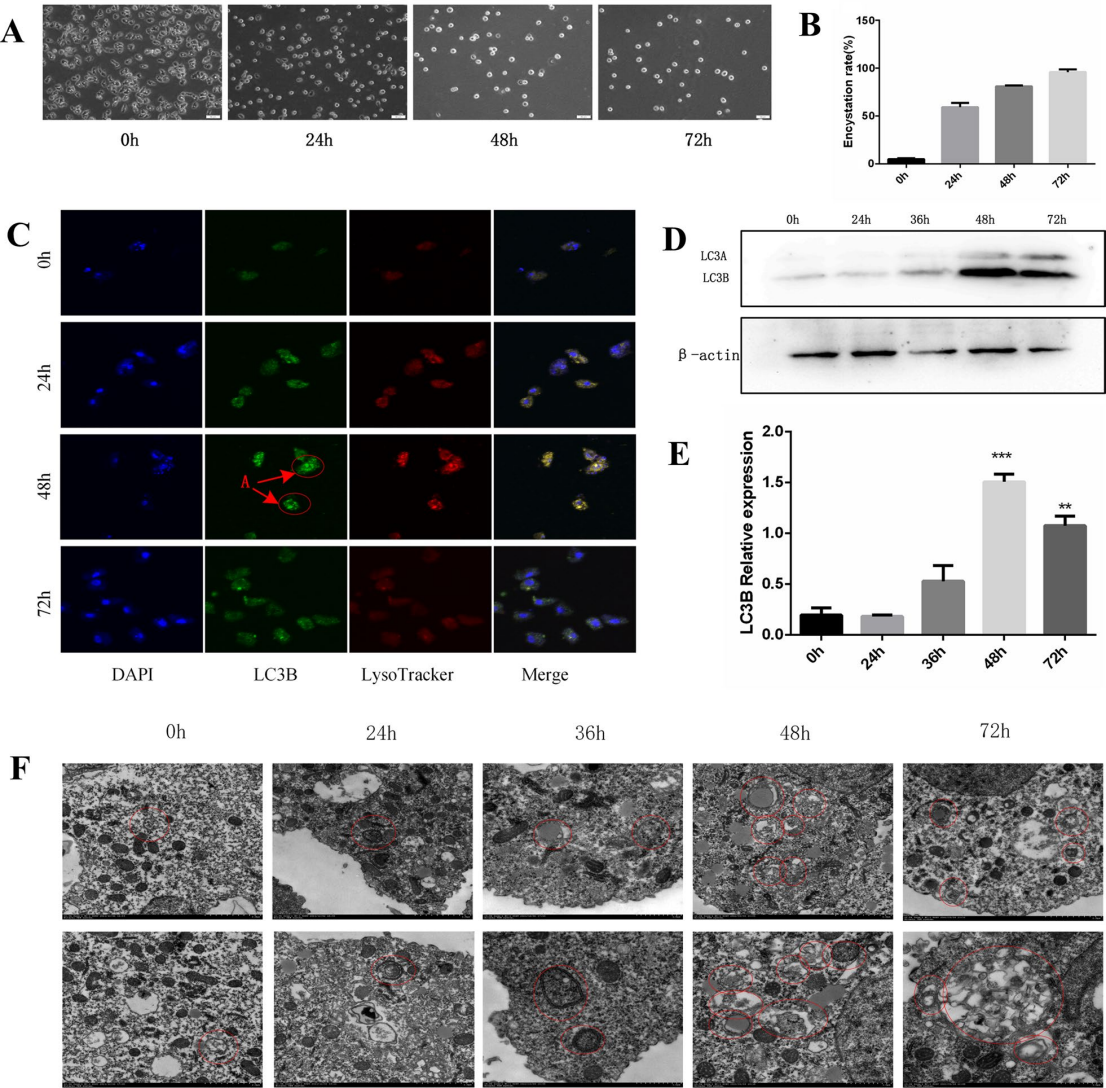
Effects of encystation on autophagy under ice bath for 0–72 h. Trophozoites were randomly divided into four time groups, and encystation was induced for 0 h, 24 h, 48 h and 72 h in an ice bath, respectively. **A** Microscopic counting on the encystation rate of trophozoites induced by ice bath for 0–72 h. **B** Effect of induction time on the encystation rate (3 fields were randomly selected, and the average of encystation rate from three fields was calculated). **C** Effects of different encystation times on autophagy fluorescence of LC3B were observed by IF assay (A, the typical fluorescent spots of autophagic vesicles formed after the occurrence of autophagy in *Acanthamoeba castellanii*). **D** Protein bands of autophagy protein LC3B during 0–72 h of encystation were detected by western blot. **E** Effects of different encystation times on protein expression of LC3B (The experiment was repeated three times. Compared to 0 h, **P < 0.01, ***P < 0.001). **F** Effect of encystation for 0–72 h on the autolysosome-like structures inside *A. castellanii* was observed by transmission electron microscopy. (The autolysosome-like structures are marked with red circles)

First, the effect of induction time on the encystation rate of *A. castellanii* was analyzed. It was found that the encystation rate of *A. castellanii* increased significantly with the extension of incubation time in an ice bath, and the encystation rates at 0, 24, 48 and 72 h were 9.21%, 60.37%, 76.98% and 98.51%, respectively (Fig. 2A, B). The fluorescence intensity of LC3B gradually increased with the extension of incubation time in the ice bath, reached the maximum at 48 h and then decreased slightly (Fig. 2C). In addition, the western blotting results showed that the protein expression of LC3B increased with the extension of encystation time, reached the maximum at 48 h and decreased slightly at 72 h (Fig. 2D, E). The autophagosomes are vacuole-like structures with double or multilayer membranes, containing cytoplasmic components, such as mitochondria, endoplasmic reticulum, ribosomes, etc. The autolysosomes have a monolayer membrane structure with degradation of cytoplasmic components, which contain acid phosphatase activity (black deposits) indicating that fusion with lysosomes has occurred [25, 26]. Electron microscopy showed that the internal structure and cell membrane were complete, and clear autolysosomes were formed. The number of autophagic vacuoles increased with the increase in encystation time and reached the maximum at 48 h (Fig. 2F). The results indicated that there was a positive correlation between autophagy and encystation in *A. castellanii*.

### Regulatory effect of *Ac*-HSP20 on encystation in *A. castellanii*

**Fig. 3:**
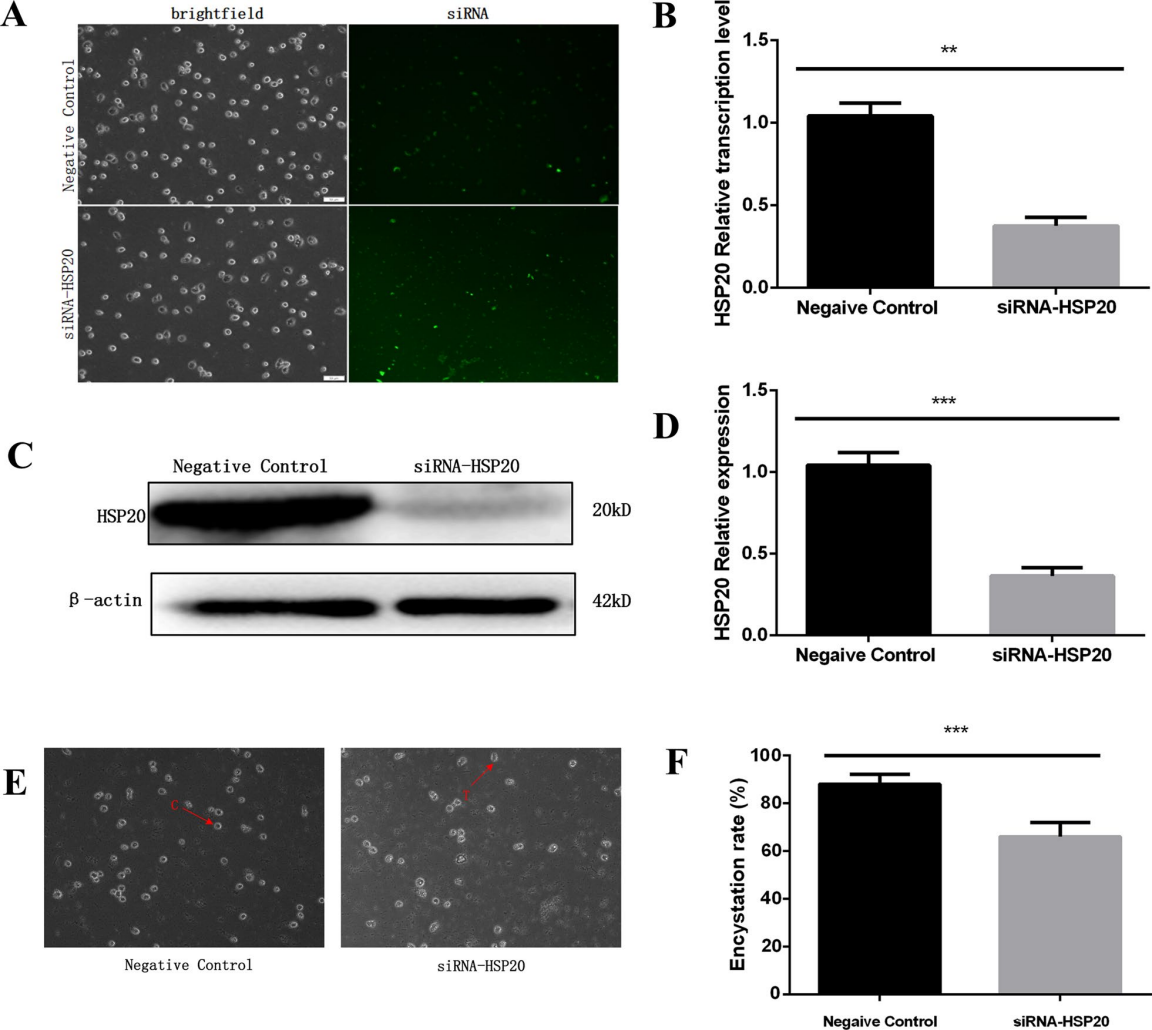
Effects of *Ac*-HSP20 knockdown on encystation in *Acanthamoeba castellanii.* Trophozoites were transfected with *Ac*-HSP20 siRNA, and encystation was induced for 48 h in an ice bath. **A** Successful transfection of siRNA was observed by fluorescence microscopy. **B** Transcriptional inhibition of *Ac*-HSP20 mRNA by siRNA was detected by qRT-PCR. (The experiment was repeated three times. **P < 0.01.) **C** Protein bands of *Ac*-HSP20 after siRNA transfection were detected by western blot. **D** Statistical analysis of the protein expression of *Ac*-HSP20. (The experiment was repeated three times. ***P < 0.001.) **E** Microscopic observation of the encystation rate after knockdown of *Ac*-HSP20 (T = trophozoite characterized by irregular spinous appearance, C = cyst characterized by regular round shape). **F** Effect of *Ac*-HSP20 knockdown on the encystation rate (three fields were randomly selected, and the average encystation rate from three fields was calculated. ***P < 0.001)

The successful transduction of *Ac*-HSP20 siRNA into *A. castellanii* trophozoites was confirmed by fluorescence staining at 6 h after siRNA treatment (Fig. 3A). Compared with the negative control group, the gene transcription level of *Ac*-HSP20 was significantly inhibited to 38.66% at 24 h (Fig. 3B) (*P* < 0.01), and the protein expression level was significantly inhibited to 32.76% at 48 h by siRNA (Fig. 3C, D) (*P* < 0.001). An ice bath was used to induce encystation for 48 h after the knockdown of *Ac*-HSP20. Compared with the negative control group, the encystation rate of the *Ac*-HSP20 knockdown group decreased from 82.77% to 63.87% (Fig. 3E, F), with statistical significance (*P* < 0.001). The results indicated that *Ac*-HSP20 had a regulatory effect on the encystation of *A. castellanii*.

### Regulatory effect of *Ac*-HSP20 on autophagy on *A. castellanii*

**Fig. 4:**
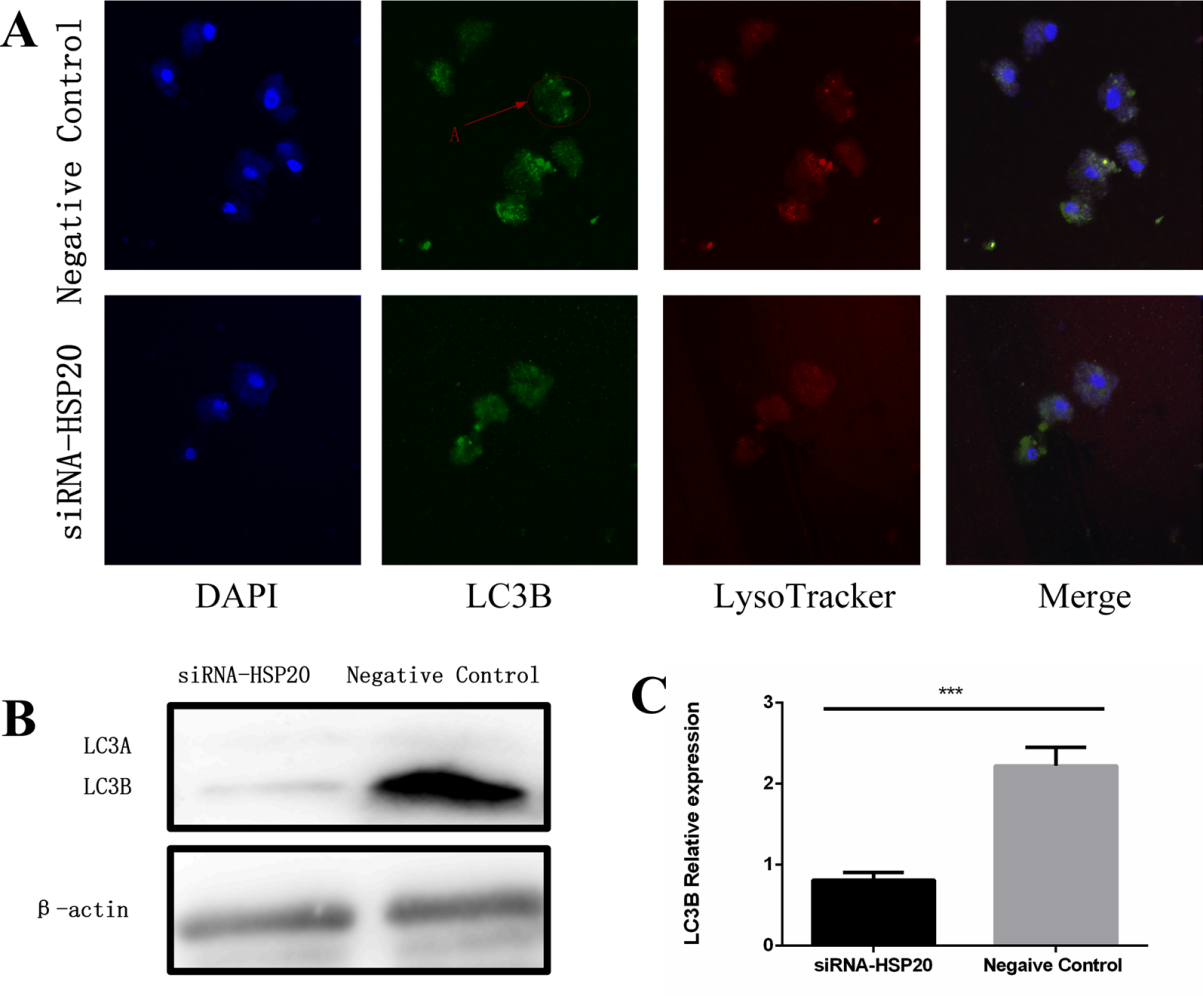
Effects of *Ac*-HSP20 knockdown on autophagy in *Acanthamoeba castellanii*. Trophozoites were transfected with *Ac*-HSP20 siRNA, and encystation was induced for 48 h in an ice bath. **A** Effects of *Ac*-HSP20 knockdown on autophagy fluorescence of LC3B were observed by IF assay (A: the typical fluorescent spots of autophagic vesicles formed after the occurrence of autophagy in *A. castellanii*). **B** Protein bands of LC3B after siRNA transfection were detected by western blot. **C** Statistical analysis on the protein expression of LC3B. (The experiment was repeated three times. ***P < 0.001)

Compared with the negative control group, LC3B fluorescence signal intensity was significantly decreased in the *Ac*-HSP20 knockdown group (Fig. 4A). In addition, the protein expression of LC3B was decreased in the knockdown group (Fig. 4B, C). The results indicated that *Ac*-HSP20 played a regulatory role in autophagy in *A. castellanii*.

### Regulatory effect of *Ac*-HSP20 on *A. castellanii* autophagy via the PI3K/AKT/mTOR signaling pathway

**Fig. 5:**
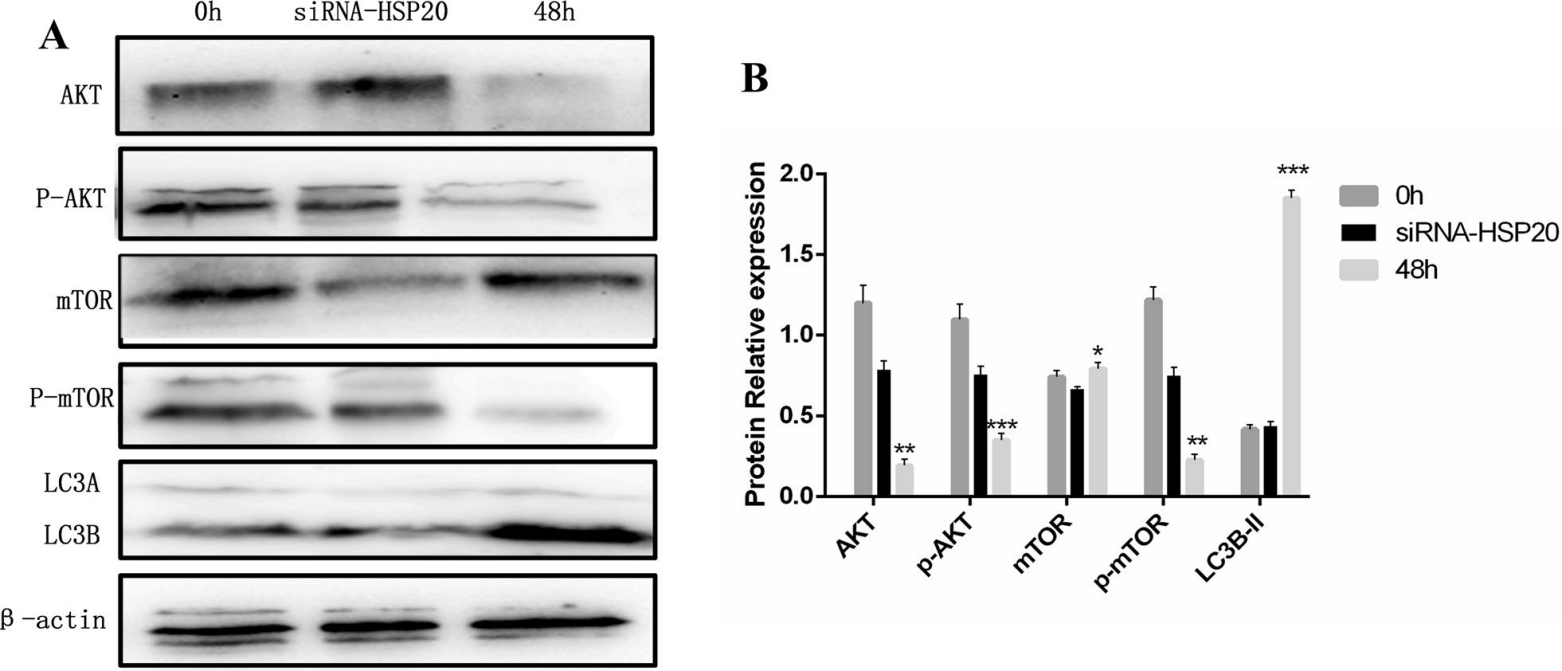
Effects of *Ac*-HSP20 on autophagy protein LC3B and autophagy-related pathway PI3K/AKT/mTOR. Control group without ice bath and *Ac*-HSP20 knockdown (0 h), *Ac*-HSP20 knockdown group with ice bath for 48 h (siRNA-HSP20), normal encystation group with ice bath for 48 h. **A** The protein bands of LC3B and PI3K/AKT/mTOR pathway-related proteins were detected by western blot. **B** Statistical analysis on the protein expressions of LC3B and PI3K/AKT/mTOR pathway-related proteins. (The experiment was repeated three times. Compared to siRNA-HSP20 group, *P < 0.05, **P < 0.01, ***P < 0.001)

To explore the mechanism of the involvement of *Ac*-HSP20 in the autophagy and encystation of *A. castellanii*, the relationship between *Ac*-HSP20 and autophagy-related pathway PI3K/AKT/mTOR was further investigated. Compared with the control group without the ice bath and knockdown, the protein expression levels of p-AKT and p-mTOR were significantly decreased, and the expression level of LC3B was increased after encystation for 48 h (Fig. 5) (*P* < 0.05, *P* < 0.01 and *P* < 0.001). The results indicated that the PI3K/AKT/mTOR pathway was negatively correlated with the occurrence of autophagy during the process of encystation in *A. castellanii*. Compared with the encystation group, in the *Ac*-HSP20 knockdown group, the protein expression levels of p-AKT and p-mTOR were significantly increased, and the expression level of LC3B was significantly decreased (Fig. 5) (*P* < 0.05, *P* < 0.01 and *P* < 0.001). These results suggested that *Ac*-HSP20 might regulate autophagy in *A. castellanii* by inhibiting the PI3K/AKT/mTOR signaling pathway, thus promoting the maturation of encystation.

**Fig. 6:**
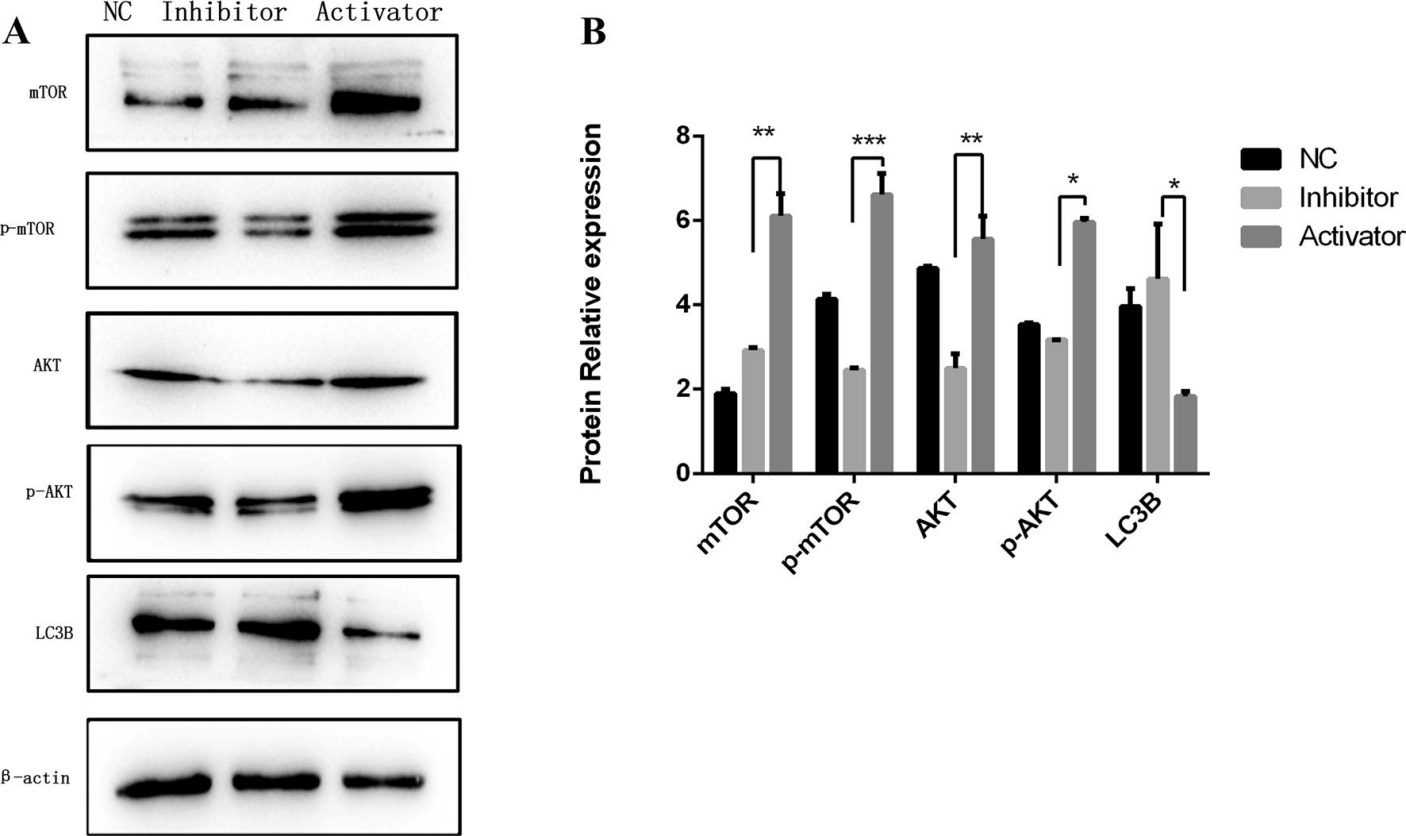
Effect of *Ac*-HSP20 on autophagy when the PI3K/AKT/mTOR pathway was inhibited and activated. Trophozoites were transfected with *Ac*-HSP20 siRNA, and encystation was induced for 48 h in an ice bath. Signal inhibitor: PI3K/mTOR inhibitor-11 (1 mmol/l). Signal activator: Recilisib (1.25 mmol/l). **A** Protein bands of LC3B and PI3K/AKT/mTOR pathway-related proteins after siRNA transfection were detected by western blot. **B** Statistical analysis on the protein expressions of LC3B and PI3K/AKT/mTOR pathway-related proteins. (The experiment was repeated three times. Compared to control group, *P < 0.05, **P < 0.01, ***P < 0.001)

Pathway inhibitors and activators were used for further validation under the condition of *Ac*-HSP20 knockdown. Compared with the negative control group, the protein expression levels of p-AKT and p-mTOR were downregulated, and the expression of LC3B was upregulated when the pathway was inhibited. Contrarily, after activation of this pathway, the expression levels of p-AKT and p-mTOR were upregulated, and the protein level of LC3B was downregulated (Fig. 6) (*P* < 0.05, *P* < 0.01, *P* < 0.001). These findings suggest that *Ac*-HSP20 promotes the autophagy and encystation of *A. castellanii* by inhibiting the PI3K/AKT/mTOR pathway.

## Discussion

Autophagy plays an important role in the encystation of *Acanthamoeba* [27, 28]. The autophagy-related proteins Atg3, Atg8 and Atg16 are highly expressed during the process of encystation in *Acanthamoeba* [29–33]. To explore the mechanism of the involvement of autophagy in the process of *A. castellanii* cyst formation, the 3-MA inhibitor was used in this study to inhibit autophagy. The encystation rate of *A. castellanii* trophozoites and fluorescence intensity of LC3B were decreased compared with the control group (Fig. 1). These results indicated that when autophagy was inhibited, the encystation of *A. castellanii* was also inhibited, demonstrating that the encystation was directly affected by autophagy. Similar studies showed that 3-MA treatment significantly lowered the encystation rate and autophagosomes in *Cryptocaryon irritans* [34]. Subsequently, the fluorescence signal intensity and protein expression of the autophagy-associated protein LC3B were determined using IF and western blotting tests after the induction of encystation for 0–72 h. The results showed that the fluorescence intensity and protein expression of LC3B increased with the induced duration of encystation and reached the maximum at 48 h of encystation (Fig. 2), indicating that autophagy occurred during the process of cyst formation. Finally, transmission electron microscopy revealed that the number of autophagic lysosomes reached the maximum when the protozoa were cooled on ice for 48 h, which supported the above results. These results indicate that autophagy is positively correlated with the encystation of *A. castellanii*.

Our previous study demonstrated that the expression of *Ac*-HSP20 was increased during the process of encystation in *A. castellanii*. *Ac*-HSP20 knockdown and antibody incubation experiments showed that the initiation of cyst formation was inhibited and delayed from 24 to 48 h, which proved that *Ac*-HSP20 was related to the initiation of encystation [22]. Since the exchange between *A. castellanii* and the external environment stops after the initiation of encystation. It is speculated that *Ac*-HSP20 can also promote the maturation of cysts by regulating autophagy and participating in cell homeostasis during the process of encystation. Therefore, the relationship between *Ac*-HSP20 and autophagy, as well as its underlying mechanism, was explored in the present study. The encystation rate of *A. castellanii* decreased when the *Ac*-HSP20 gene was knocked down using siRNA transfection (Fig. 3). The subsequent detection of autophagy levels showed that silencing *Ac*-HSP20 could reduce the protein expression of LC3B and the number of autophagosomes (Fig. 4). These results indicated that *Ac*-HSP20 had a positive regulatory effect on autophagy in *A. castellanii*, and it was involved in maintaining of cell homeostasis to promote the maturation of encystation by regulating autophagy.

The PI3K/AKT/mTOR signaling pathway plays a key role in the regulation of autophagy. The expression levels of autophagy-related proteins increase when the PI3K/AKT/mTOR pathway is inhibited [35]. mTOR, the main target of the autophagy signaling pathway, is activated through AKT phosphorylated by PI3K, which promotes the interaction of mTOR with autophagy-related factor serine kinase 1 and ultimately triggers autophagy [36]. The crosstalk between AKT and mTOR is involved in molecular apoptosis and autophagy. HSPs, as molecular chaperones, are essential for the proper functioning of AKT. For example, HSP90 forms the chaperon-substrate protein complex, and reduced HSP-AKT binding leads to AKT inactivation [37]. The reduction in mTOR levels by RNA interference leads to increased sensitivity to heat shock [38]. Studies have shown that the autophagy of HL-1 cardiomyocytes was promoted by regulating AKT and mTOR via transporting HSP70 [39]. The present study further explored the relationship between the autophagy of *A. castellanii* and this pathway, as well as the relevant mechanism of *Ac*-HSP20 participation. The results showed that the expression levels of *Ac*-HSP20 and LC3B increased and that the expression levels of the pathway proteins p-AKT and p-mTOR decreased after 48 h of encystation (Fig. 5). However, the expression levels of p-AKT and p-mTOR increased, while the expression of LC3B decreased under the silencing of the *Ac*-HSP20 gene (Fig. 5). These results suggested that *Ac*-HSP20 might regulate autophagy by inhibiting the PI3K/AKT/mTOR signaling pathway.

To clarify the relationship between *Ac*-HSP20 and the PI3K/AKT/mTOR signaling pathway, and its mechanism in regulating autophagy and promoting encystation, a PI3K inhibitor and a PI3K/AKT activator were applied to *A. castellanii* with *Ac*-HSP20 knocked down to observe the changes in the expression levels of the pathway proteins and autophagy proteins. It was found that the protein expression of LC3B increased when the pathway was inhibited, while it decreased when the pathway was activated (Fig. 6). The results further demonstrated that *Ac*-HSP20 regulated autophagy in *A. castellanii* through the PI3K /AKT /mTOR signaling pathway. The findings of the present study reveal for the first time to our knowledge that HSP20 regulates autophagy to maintain the homeostasis of *A. castellanii*, thus promoting the maturation and stability of encystation by inhibiting the PI3K/AKT/mTOR signaling pathway.

## Conclusions

In summary, this study found that autophagy had a positive effect on the encystation of *A. castellanii*, and the autophagy level reached its maximum at 48 h of encystation. The underlying mechanism was fact that *Ac*-HSP20 regulated autophagy to maintain the homeostasis of *A. castellanii* and to promote encystation by inhibiting the PI3K/AKT/mTOR signaling pathway. Compared with previous research, new dynamic and systematic analysis methods were used in this study to explore the relationship between autophagy and encystation. Dynamic molecular changes at different time points of encystation were analyzed. The regulatory effects of HSP20 on autophagy and encystation of *A. castellanii* were revealed, and the PI3K/AKT/mTOR signaling pathway was found to be inhibited in the regulation of autophagy to promote the encystation. This study provides a new perspective to elucidate the molecular mechanism underlying the regulation of *Acanthamoeba* encystation as well as a theoretical basis for the development of anti-*Acanthamoeba* therapeutic drugs.

## Data Availability

The data supporting the findings of this study are available within the article.

## Abbreviations

*A. castellanii*: *Acanthamoeba castellanii*
HSP: Heat shock protein
*Ac*-HSP20: Heat shock protein 20 of *A. castellanii*
3-MA: 3-Methyladenine
SDS: Sodium dodecyl sulfate
IF: Immunofluorescence
TEM: Transmission electron microscopy
PI3K: Phosphatidylinositol 3-kinase
AKT: RAC-alpha serine/threonine-protein kinase
mTOR: Mammalian target of rapamycin
LC3B: Microtubule-associated proteins 1A/1B light chain 3B
